# Supplementary material for: Synaptic density affects clinical severity via network dysfunction in syndromes associated with frontotemporal lobar degeneration
Source: Nat Commun. 2023 Dec 20;14:8458. doi: 10.1038/s41467-023-44307-7 (PMC10730886; doi:10.1038/s41467-023-44307-7)
Supplement: Supplementary file 1 — Supplementary Information [file 41467_2023_44307_MOESM1_ESM.pdf]

Supplementary Information

Supplementary Methods

Group differences in [^{11}C]UCB-J BP_{ND} with voxelwise regression of grey matter segmentations

We tested for voxelwise differences between patients and controls in [^{11}C]UCB-J BP_{ND} with grey matter segmentations included as a covariate of no interest. Warped and modulated grey matter segmentations in MNI spaced were derived using the Computational Anatomy Toolbox 12 (https://neuro-jena.github.io/cat) in SPM 12 ((https://www.fil.ion.ucl.ac.uk/spm/software/spm12). Differences between healthy controls and participants with FTLD-associated syndromes were assessed in a general linear model in PALM ('Permutation Analysis of Linear Models') with 1000 permutations and family-wise error P value correction. The -evperdat option was used to allow for voxelwise regression. Total intracranial volume was included as a covariate of no interest.

Source-based synaptometry following spatial regression of grey matter segmentations

To test if atrophy was an important determinant of the observed pattern of differences in [^{11}C]UCB-J BP_{ND} participant component loadings, we repeated independent component analysis following spatial regression of grey matter tissue probability maps from the smoothed and normalized partial volume corrected [^{11}C]UCB-J BP_{ND} maps.[1] Grey matter probability maps were derived as above and smoothed with an 8mm FWHM Gaussian kernel. Spatial regression of the concatenated grey matter segmentations against the [^{11}C]UCB-J BP_{ND} maps was performed using FSL's fsl_glm function. Source-based synaptometry with model order 10 was performed as per the primary analysis. Components were matched to the original components using spatial cross-correlation.

Supplementary Results

Weighted degree and [^{11}C]UCB-J BP_{ND} with different parcellations

We fit the same linear mixed-effects models using the full (i.e. not reparcelled) Hammersmith Atlas, with cortical parcels within a grey matter mask. Weighted degree was associated with [^{11}C]UCB-J BP_{ND} in patients (Standardised Beta 0.19, P=1x10^{-11}) but not in control participants (Standardised Beta 0.0 P=0.90). The group-by-[^{11}C]UCB-J BP_{ND} interaction in a refitted model with all participants was significant ([^{11}C]UCB-J BP_{ND}*Group Standardised

Beta 0.076, $P=9x10^{-5}$).The relationship between $[^{11}C]$UCB-J BP_{ND} and weighted degree was also observed in each FTLD syndrome individually (bvFTD Standardised Beta 0.19 $P=3x10^{-5}$; CBS Standardised Beta 0.14 P=0.002; PSP Standardised Beta 0.25, $P<9x10^{-11}$).

We repeated the models using the Brainnetome parcellation. Weighted degree was associated with $[^{11}C]$UCB-J BP_{ND} in patients (Standardised Beta 0.14, $P<2x10^{-16}$) and also in control participants (Standardised Beta 0.05 P=0.004). The group-by-$[^{11}C]$UCB-J BP_{ND} interaction in a refitted model with all participants was significant ($[^{11}C]$UCB-J BP_{ND}*Group Standardised Beta 0.034, P=0.004). The relationship between $[^{11}C]$UCB-J BP_{ND} and weighted degree was also observed in each FTLD syndrome individually (bvFTD Standardised Beta 0.17 $P<2x10^{-16}$; CBS Standardised Beta 0.12 $P=1x10^{-11}$; PSP Standardised Beta 0.15, $P<2x10^{-16}$).

Source-based synaptometry following spatial regression of grey matter images

We repeated source-based synaptometry with model order 10 on the partial volume corrected normalised $[^{11}C]$UCB-J BP_{ND} maps following spatial regression of grey matter tissue probability maps. The resulting components were matched using spatial cross-correlation to the six components that differed between-groups in our primary analysis. Participant loadings for the matched component were well correlated (mean Pearson's R 0.81, range 0.58-0.96). We found between-group differences in these components with a highly similar pattern of group differences as found using $[^{11}C]$UCB-J BP_{ND} maps without spatial regression of grey matter tissue probability maps (Supplementary Fig. 6), suggesting that atrophy or partial volume effects are not key determinants of these results.

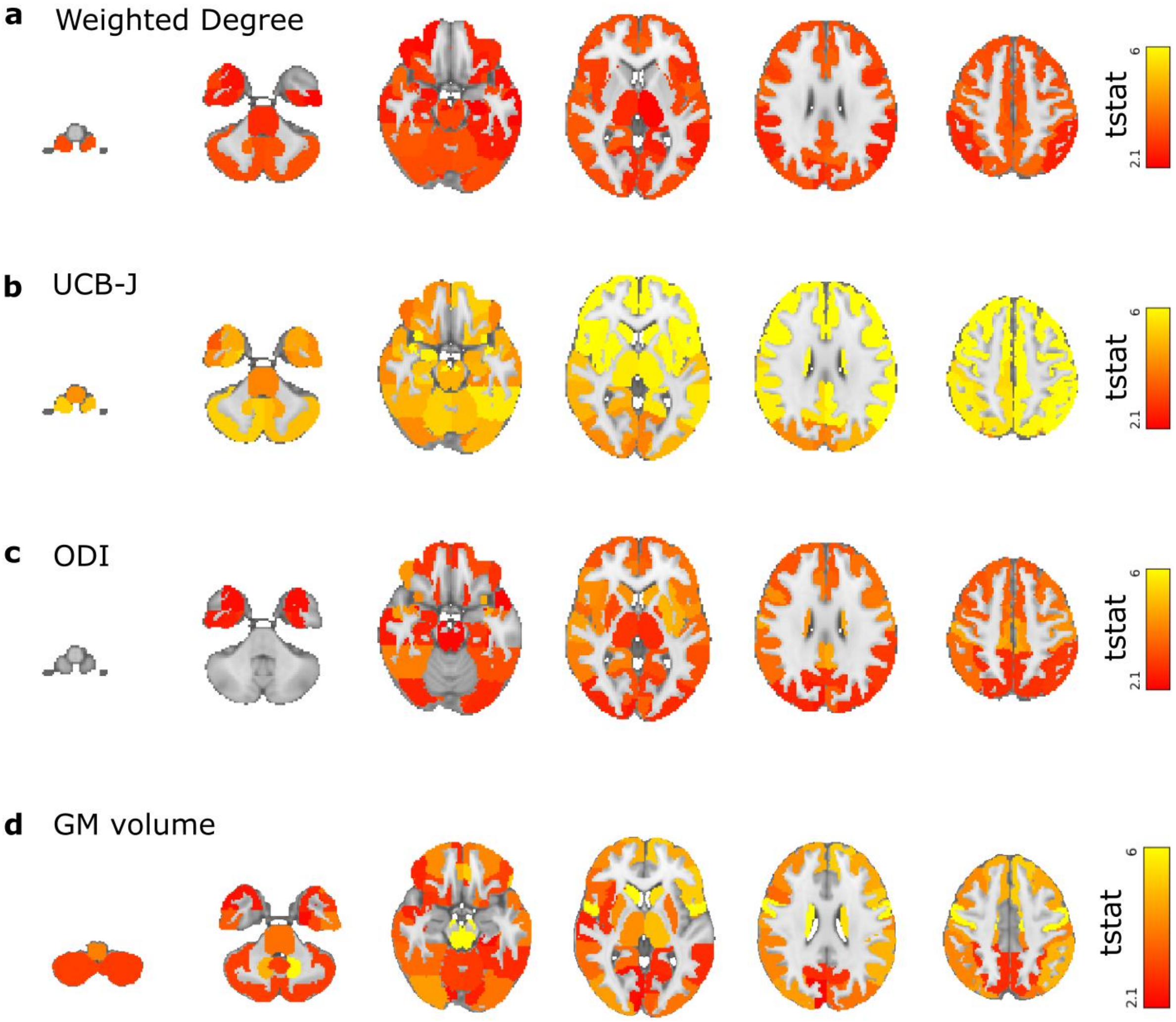

Supplementary Figure 1: Regional reductions by modality in patients compared to healthy controls.

Regional reductions in patients compared to healthy controls in **a)** weighted degree (controls n=24, patients n=55), **b)** [^{11}C]UCB-J BP_{ND} (controls n=24, patients n=55), **c)** orientation dispersion index (ODI) (controls n=23, patients n=52), and **d)** grey matter (GM) volume (controls n=24, patients n=55). All parcels shown are significantly different after FDR-correction for multiple comparisons across regions. Age and sex were included as covariates of no interest, plus total intracranial volume for grey matter volume and mean DVARS for weighted degree. Source data are provided as a Source Data file.

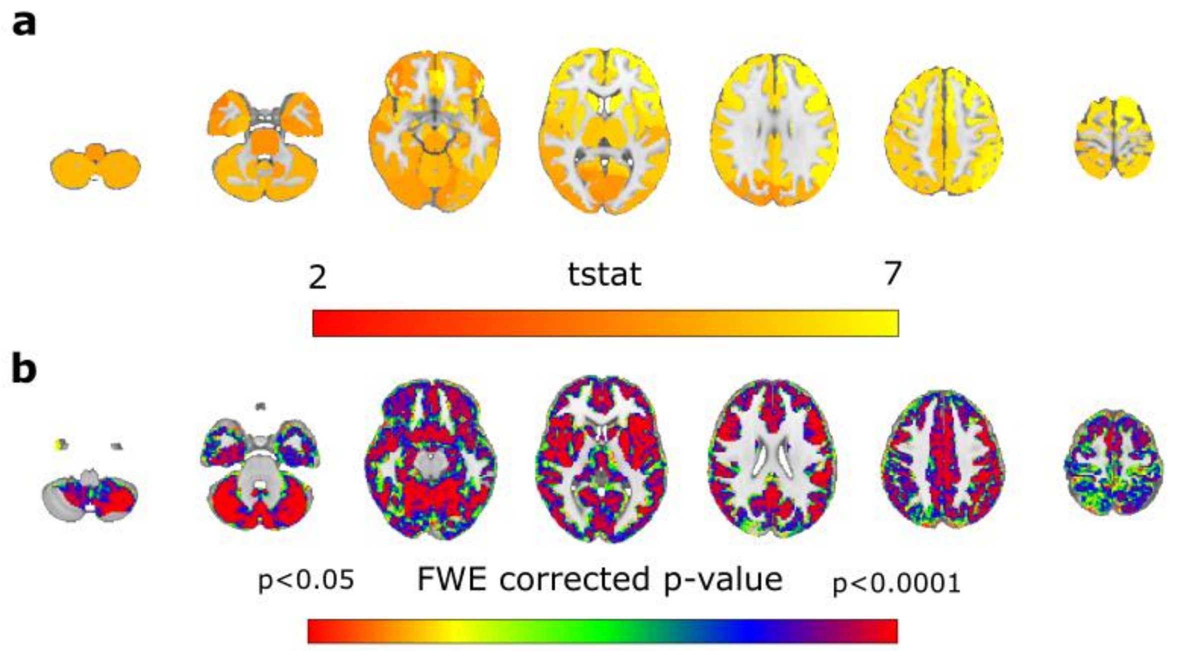

Supplementary Figure 2: Differences in UCBJ BP_{ND} with grey matter volume as a covariate of no interest

a) Regional and **b)** voxelwise differences in weighted degree between healthy controls (n=24) and patients (n=55) in [^{11}C]UCB-J BP_{ND} with grey matter volumes/ brainstem total volumes included as a covariate of no interest. All regional parcels shown are significantly different after FDR-correction for multiple comparisons across regions. Voxelwise statistics calculated using 1000 permutations within a grey matter mask with family-wise error (FWE) across voxels. Source data are provided as a Source Data file.

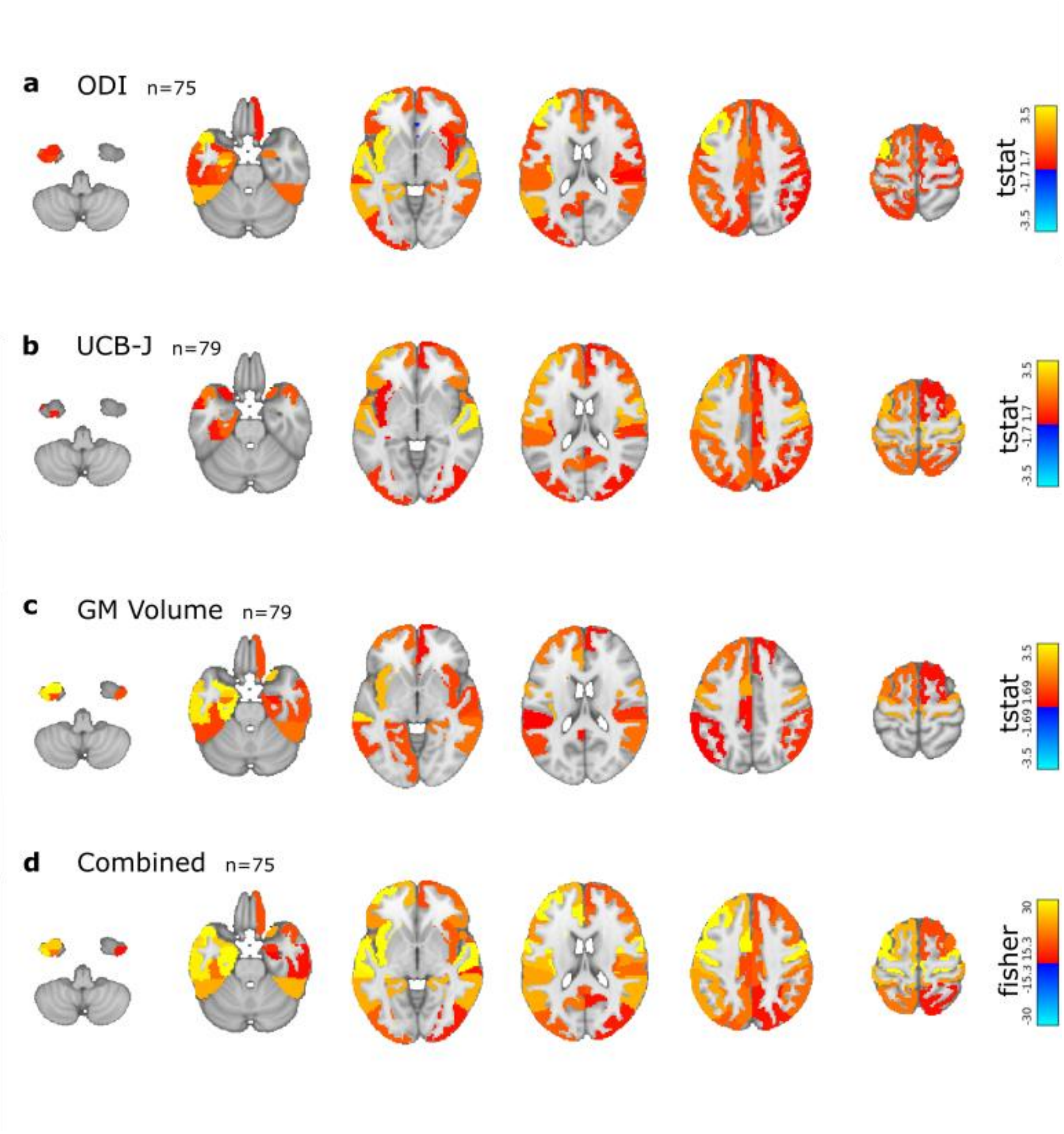

Supplementary Figure 3: Uncorrected regional significant associations between weighted degree and other modalities

Regional relationship between weighted degree and **a)** orientation dispersion index (ODI), **b)** [^{11}C]UCB-J BP_{ND}, **c)** grey matter (GM) volumes**,** and **d)** combined modalities using non-parametric combination testing with 10000 permutations. All parcels shown are uncorrected significant $p<0.05$. Age, sex, and mean DVARS were covariates of no interest, plus total intracranial volume where volumetric measures were included. Source data are provided as a Source Data file.

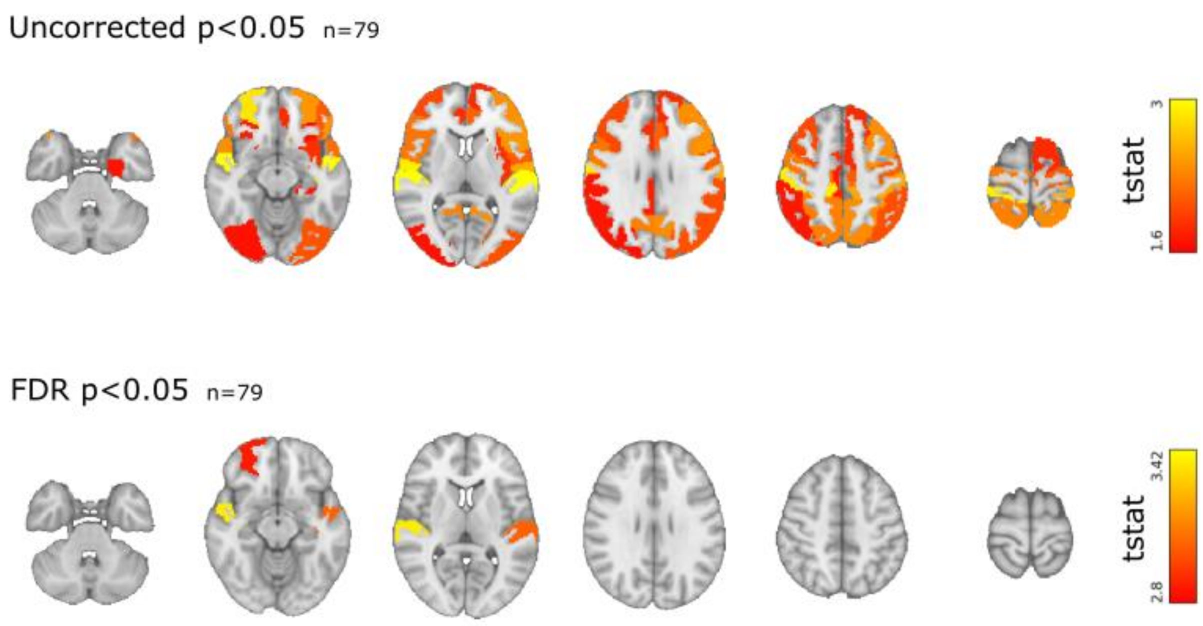

Supplementary Figure 4: Regional relationship between weighted degree and [^{11}C]UCB-J BP_{ND} with regional grey matter volume as a covariate of no interest

Regional relationship between weighted degree and [^{11}C]UCB-J BP_{ND} with regional grey matter volume as a covariate of no interest in addition to age, sex, fMRI motion, and total intracranial volume. FDR: false discovery rate. Source data are provided as a Source Data file.

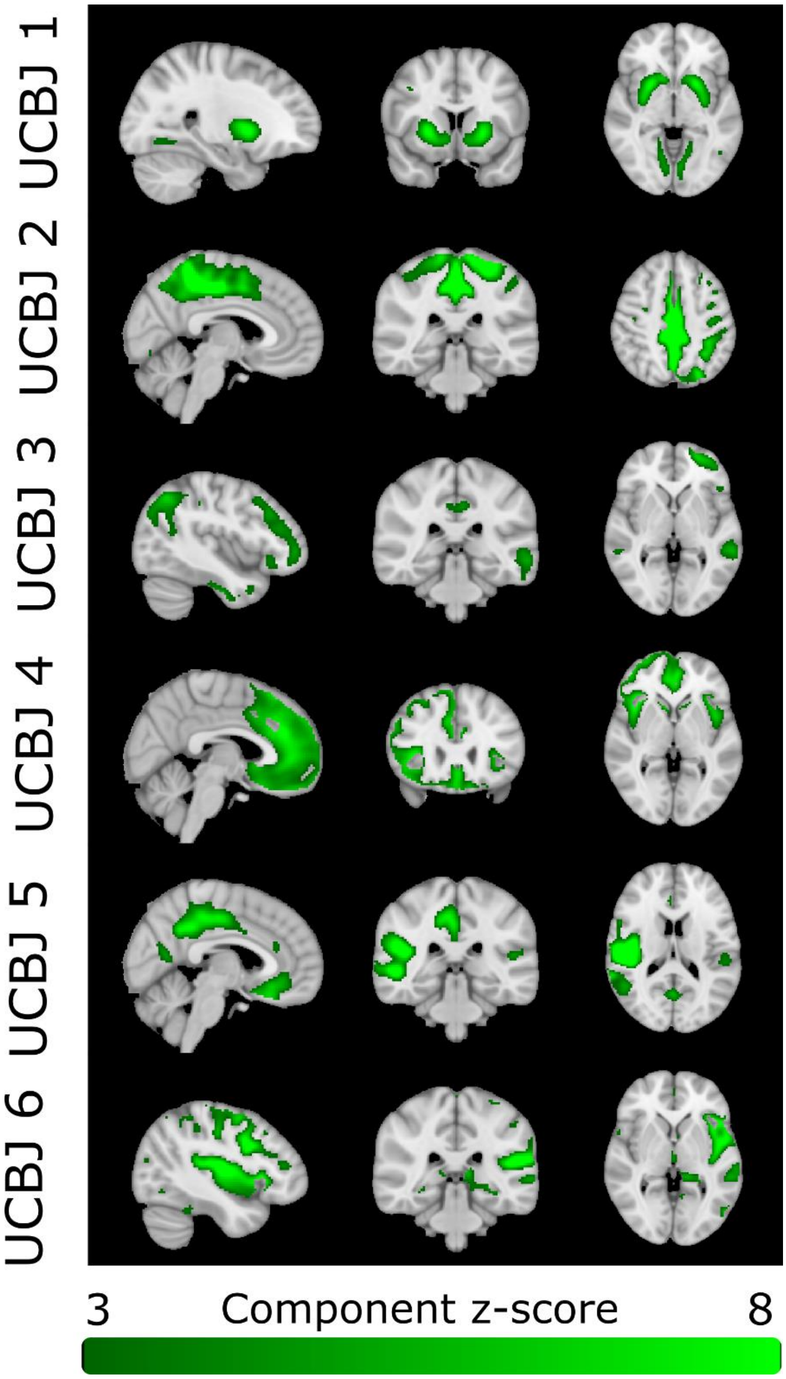

Supplementary Figure 5: [^{11}C]UCB-J BP$_{ND}$ components that show post-correction between group differences

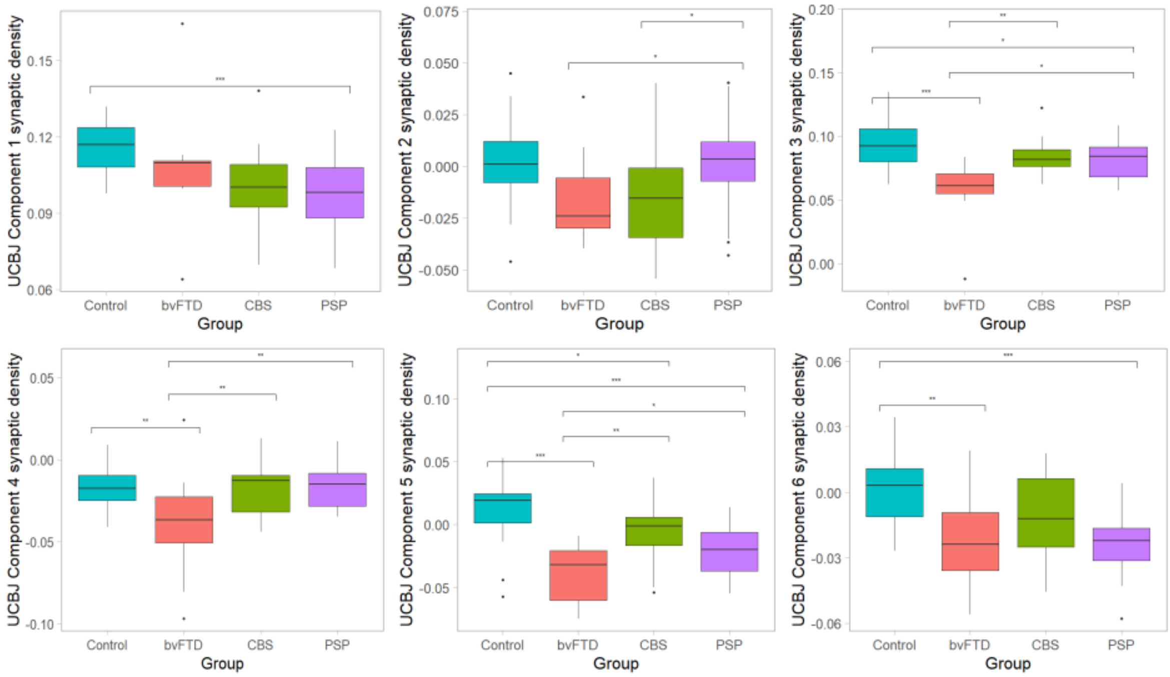

Control n=24 bvFTD n=10 CBS n=16 PSP n=29

Supplementary Figure 6: Participant loadings by group for components derived using source based synaptometry from [^{11}C]UCB-J BP_{ND} maps following spatial regression of grey matter tissue probability maps.

Box and whisker plots show median, inter-quartile range, with whiskers extending from the hinge to the largest/smallest point at most 1.5 times the interquartile range from the hinge. Significance brackets (* p< 0.05, ** p<0.01, *** p<0.001) represent P values from Tukey adjusted post-hoc pairwise comparisons. Component 1 PSP < Control P=0.0008; Component 2 bvFTD < PSP P=0.027, CBS < PSP P=0.05; Component 3 bvFTD < Control P<0.0001, bvFTD < CBS P=0.0073, bvFTD < PSP P=0.014, PSP < Control P=0.026, Component 4 bvFTD < Control P=0.0049, bvFTD < CBS P=0.0076, bvFTD < PSP P=0.0029; Component 5 bvFTD < Control P<0.0001, bvFTD < CBS P=0.0018, bvFTD < PSP P=0.012, CBS < Control P=0.011, PSP < Control P<0.0001; Component 6 bvFTD < Control P=0.0034, PSP < Control P < 0.0001. Age and sex were included as covariates of no interest. Source data are provided as a Source Data file.

Supplementary Table 1. Medications of note used at [^{11}C]UCB-J BP_{ND} scanning session for participants with frontotemporal lobar degeneration associated syndromes

	**bvFTD**	**CBS**	**PSP**
Anti-Parkinsonian Medications		Amantadine n=5<br>Levo-dopa n=5	Amantadine n=7<br>Levo-dopa n=10
Selective serotonin uptake inhibitors	Citalopram/ Sertraline/ Fluoxetine n=5	Citalopram/ Sertraline /Fluoxetine n=3	Citalopram/ Fluoxetine n=10
Behavioural management	Trazadone/ Risperidone n=4		
Statin	Atorvastatin n=2	Atorvastatin/ Simvastatin n=4	Atorvastatin n=4
Anti-hypertensives	Lisinopril/ Ramipril n=2	Ramipril/ Losartan n=3	Amlodipine/ Ramipril/ Lercanidipine/ Losartan/ Lisinopril n=8
Anti-platelet agents/anti-coagulants		Aspirin / Warfarin n=5	Apixaban/ Rivaroxaban/ Clopidogrel/ Aspirin n=5

bvFTD behavioural variant frontotemporal dementia; CBS corticobasal syndrome, PSP progressive supranuclear palsy

Supplementary Table 2. Regional differences in [^{11}C]UCB-J BP_{ND} between patient groups

Region	F value	P	FDR P	*Post-hoc* tests
Cingulate gyrus anterior part L	8.1	0.00086	0.068	bvFTD < PSP P=0.026<br>bvFTD < CBS P=0.0005
Cingulate gyrus anterior part R	6.3	0.0036	0.094	bvFTD < PSP P=0.044<br>bvFTD < CBS P=0.002
Posterior orbital gyrus R	5.9	0.0052	0.094	bvFTD < PSP P=0.039<br>bvFTD < CBS P=0.004
Middle frontal gyrus L	5.7	0.0061	0.094	bvFTD < PSP P=0.027<br>bvFTD < CBS P=0.005
Anterior temporal lobe medial part R	5.6	0.0065	0.094	bvFTD < PSP P=0.018<br>bvFTD < CBS P=0.006
Pallidum R	5.3	0.0084	0.094	CBS > PSP P=0.016
Nucleus Accumbens R	4.9	0.011	0.094	bvFTD < CBS P=0.008
Subgenual frontal cortex L	4.9	0.011	0.094	bvFTD < CBS P=0.008
Superior frontal gryus L	4.9	0.012	0.094	bvFTD < CBS P=0.008
Presubgenual frontal cortex L	4.9	0.012	0.094	bvFTD < CBS P=0.009
Insula R	4.5	0.016	0.11	bvFTD < PSP P=0.045<br>bvFTD < CBS P=0.013
Inferior frontal gyrus L	4.5	0.017	0.11	bvFTD < CBS P=0.012
Subgenual frontal cortex R	4.4	0.018	0.11	bvFTD < CBS P=0.013
Nucleus Accumbens L	4.3	0.019	0.11	bvFTD < CBS P=0.014
Subcallosal area L	3.9	0.026	0.13	bvFTD < CBS P=0.020
Anterior orbital gyrus R	3.8	0.03	0.13	bvFTD < CBS P=0.023
Presubgenual frontal cortex R	3.7	0.033	0.13	bvFTD < CBS P=0.029
Middle frontal gyrus R	3.5	0.038	0.13	bvFTD < CBS P=0.031
Medial orbital gyrus R	3.4	0.041	0.13	bvFTD < CBS P=0.032
Amygdala R	3.4	0.041	0.13	bvFTD < PSP P=0.045
Medial orbital gyrus L	3.4	0.042	0.13	bvFTD < CBS P=0.033
Posterior orbital gyrus L	3.4	0.042	0.13	bvFTD < CBS P=0.034
Inferior frontal gyrus R	3.4	0.043	0.13	bvFTD < CBS P=0.036
Lateral orbital gyrus L	3.4	0.043	0.13	
Superior frontal gyrus R	3.4	0.043	0.13	bvFTD < CBS P=0.033
Anterior temporal lobe lateral part R	3.1	0.056	0.16	
Anterior temporal lobe medial part R	3.0	0.059	0.16	
Fusiform gyrus R	3.0	0.06	0.16	
Subcallosal area R	3.0	0.06	0.16	
Hippocampus R	3.0	0.061	0.16	
Middle and inferior temporal gyrus L	2.9	0.064	0.16	
Parahippocampal and ambient gyri R	2.9	0.067	0.16	

Anterior temporal lobe lateral part R	2.8	0.068	0.16	
Fusiform gyrus L	2.7	0.079	0.18	
Superior temporal gyrus anterior part L	2.4	0.097	0.22	
Superior temporal gyrus central part L	2.4	0.1	0.22	
Middle and inferior temporal gyrus R	2.3	0.11	0.23	
Superior parietal gyrus L	2.3	0.11	0.23	
Medulla	2.2	0.12	0.24	
Cingulate gyrus posterior part L	2.1	0.13	0.25	
Substantia nigra R	2.1	0.13	0.25	
Caudate Nucleus R	2.1	0.13	0.25	
Insula L	2.0	0.15	0.28	
Caudate Nucleus L	1.9	0.16	0.28	
Superior temporal gyrus central part R	1.8	0.17	0.3	
Posterior temporal lobe L	1.8	0.18	0.31	
Cingulate gyrus posterior part L	1.6	0.21	0.36	
Anterior orbital gyrus L	1.6	0.22	0.36	
Pallidum L	1.5	0.22	0.36	
Inferiolateral remainder of parietal lobe L	1.4	0.25	0.4	
Superior temporal gyrus anterior part R	1.4	0.27	0.41	
Postcentral gyrus R	1.2	0.3	0.45	
Lateral orbital gyrus R	1.2	0.3	0.45	
Lateral remainder of occipital lobe L	1.2	0.32	0.46	
Postcentral gyrus L	1.2	0.32	0.46	
Midbrain	1.2	0.32	0.46	
Putamen R	1.1	0.33	0.46	
Superior parietal gyrus R	1.0	0.37	0.51	
Thalamus R	1.0	0.38	0.51	
Inferiolateral remainder of parietal lobe R	0.9	0.42	0.56	
Precentral gyrus L	0.8	0.44	0.57	
Cuneus L	0.8	0.46	0.59	
Lingual gyrus L	0.7	0.48	0.6	
Thalamus L	0.7	0.49	0.6	
Putamen L	0.7	0.51	0.61	
Lingual gyrus R	0.7	0.51	0.61	
Cerebellum dentate L	0.6	0.58	0.67	

Parahippocampal and ambient gyri L	0.6	0.58	0.67	
Cerebellum gm L	0.5	0.61	0.69	
Posterior temporal lobe R	0.5	0.61	0.69	
Substantia nigra L	0.5	0.62	0.69	
Precentral gyrus R	0.4	0.64	0.71	
Cerebellum dentate R	0.4	0.68	0.74	
Cuneus R	0.4	0.69	0.74	
Amygdala L	0.4	0.7	0.74	
Hippocampus L	0.2	0.79	0.82	
Lateral remainder of occipital lobe R	0.2	0.81	0.83	
Pons	0.2	0.85	0.87	
Cerebellum gm R	0.1	0.88	0.88	

Post-hoc tests only performed where there is an uncorrected group difference in an ANCOVA with age and sex as covariates of no interest. Only significant results after Tukey adjustment of p-values with two-sided testing are shown. There were no post-correction significant differences between groups.

Supplementary Table 3. Regional differences in weighted degree between patient groups

Region	F value	P	FDR P	*Post-hoc* tests
Anterior temporal lobe lateral part L	4.0	0.024	0.89	bvFTD < CBS P=0.019
Substantia nigra R	3.7	0.031	0.89	bvFTD < CBS P=0.025
Superior temporal gyrus anterior part L	2.8	0.071	0.89	
Presubgenual frontal cortex L	2.4	0.1	0.89	
Inferior frontal gyrus L	2.3	0.11	0.89	
Caudate Nucleus L	2.2	0.12	0.89	
Subgenual frontal cortex L	2.0	0.14	0.89	
Anterior temporal lobe lateral part R	1.9	0.16	0.89	
Posterior orbital gyrus L	1.8	0.18	0.89	
Middle and inferior temporal gyrus L	1.8	0.18	0.89	
Cerebellum dentate L	1.7	0.19	0.89	
Anterior orbital gyrus R	1.7	0.19	0.89	
Anterior temporal lobe medial part R	1.7	0.19	0.89	
Anterior orbital gyrus L	1.7	0.19	0.89	
Anterior temporal lobe medial part L	1.5	0.22	0.89	
Caudate Nucleus R	1.5	0.24	0.89	
Inferior frontal gyrus R	1.5	0.24	0.89	
Fusiform gyrus L	1.5	0.24	0.89	
Cingulate gyrus anterior part L	1.4	0.25	0.89	
Posterior temporal lobe L	1.3	0.28	0.89	
Lateral orbital gyrus R	1.3	0.29	0.89	
Subgenual frontal cortex R	1.3	0.29	0.89	
Posterior orbital gyrus R	1.2	0.31	0.89	
Putamen L	1.1	0.34	0.89	
Middle frontal gyrus L	1.1	0.35	0.89	
Superior temporal gyrus central part R	1.0	0.36	0.89	
Lateral orbital gyrus L	1.0	0.37	0.89	
Superior frontal gryus L	1.0	0.38	0.89	
Medial orbital gyrus L	1.0	0.39	0.89	
Hippocampus L	0.9	0.4	0.89	
Superior parietal gyrus L	0.9	0.4	0.89	
Presubgenual frontal cortex L	0.9	0.41	0.89	

Insula R	0.9	0.42	0.89	
Amygdala L	0.8	0.45	0.89	
Precentral gyrus R	0.8	0.47	0.89	
Superior temporal gyrus central part L	0.7	0.48	0.89	
Cingulate gyrus posterior part R	0.7	0.48	0.89	
Putamen R	0.7	0.49	0.89	
Middle and inferior temporal gyrus R	0.7	0.5	0.89	
Insula L	0.6	0.53	0.89	
Midbrain	0.6	0.53	0.89	
Postcentral gyrus L	0.6	0.54	0.89	
Medial orbital gyrus R	0.6	0.54	0.89	
Cingulate gyrus anterior part R	0.6	0.55	0.89	
Lateral remainder of occipital lobe L	0.6	0.56	0.89	
Precentral gyrus L	0.6	0.57	0.89	
Hippocampus R	0.6	0.57	0.89	
Superior temporal gyrus anterior part R	0.5	0.58	0.89	
Medulla	0.5	0.59	0.89	
Inferiolateral remainder of parietal lobe L	0.5	0.6	0.89	
Pons	0.5	0.6	0.89	
Lateral remainder of occipital lobe R	0.5	0.63	0.89	
Substantia nigra L	0.5	0.63	0.89	
Cuneus R	0.5	0.64	0.89	
Lingual gyrus R	0.4	0.65	0.89	
Parahippocampal and ambient gyri L	0.4	0.66	0.89	
Pallidum L	0.4	0.67	0.89	
Superior frontal gyrus R	0.4	0.67	0.89	
Superior parietal gyrus R	0.4	0.67	0.89	
Cuneus L	0.4	0.68	0.89	
Amygdala R	0.4	0.69	0.89	
Posterior temporal lobe R	0.4	0.7	0.89	
Nucleus Accumbens L	0.3	0.74	0.9	
Middle frontal gyrus R	0.3	0.74	0.9	
Postcentral gyrus R	0.3	0.74	0.9	
Thalamus L	0.3	0.75	0.9	
Nucleus Accumbens R	0.2	0.8	0.93	
Subcallosal area L	0.2	0.81	0.93	
Inferiolateral remainder of parietal lobe R	0.2	0.81	0.93	
Cerebellum dentate R	0.2	0.83	0.93	

Lingual gyrus L	0.2	0.84	0.93	
Fusiform gyrus R	0.1	0.88	0.95	
Cingulate gyrus posterior part L	0.1	0.89	0.95	
Thalamus R	0.1	0.89	0.95	
Cerebellum gm L	0.0	0.96	0.98	
Subcallosal area R	0.0	0.96	0.98	
Parahippocampal and ambient gyri R	0.0	0.97	0.98	
Cerebellum gm R	0.0	0.97	0.98	
Pallidum R	0.0	0.98	0.98	

Post-hoc tests only performed where there is an uncorrected group difference in an ANCOVA with age, in-scanner motion and sex as covariates of no interest. Only significant results after Tukey adjustment of p-values with two-sided testing are shown. There were no post-correction significant differences between groups.

Supplementary Table 4. Regional differences in orientation dispersion index between patient groups

Region	F value	P	FDR P	*Post-hoc* tests
Anterior temporal lobe lateral part R	21.0	$3x10^{-7}$	0.000014	bvFTD < CBS P= $6x10^{-7}$, bvFTD < PSP P= $9 x10^{-7}$
Subgenual frontal cortex L	20.7	$4x10^{-7}$	0.000014	bvFTD < PSP P= $5 x10^{-7}$, bvFTD < CBS P= $2 x10^{-6}$
Medial orbital gyrus R	19.5	$7 x10^{-7}$	0.000014	bvFTD < CBS P=$6 x10^{-7}$, bvFTD < PSP P=$7 x10^{-6}$
Insula R	18.5	$1 x10^{-6}$	0.000014	bvFTD < CBS P= $7 x10^{-7}$, bvFTD < PSP P= $4 x10^{-5}$
Middle and inferior temporal gyrus R	18.5	$1 x10^{-6}$	0.000014	bvFTD < CBS P= $2 x10^{-6}$, bvFTD < PSP P= $4 x10^{-6}$
Presubgenual frontal cortex L	18.2	$1 x10^{-6}$	0.000014	bvFTD < CBS P= $1 x10^{-6}$, bvFTD < PSP P= $1 x10^{-5}$
Parahippocampal and ambient gyri L	18.2	$1 x10^{-6}$	0.000014	bvFTD < CBS P= $1 x10^{-6}$, bvFTD < PSP P= $8 x10^{-6}$
Cingulate gyrus anterior part L	18.2	$1 x10^{-6}$	0.000014	bvFTD < CBS P= $2 x10^{-6}$, bvFTD < PSP P= $4x10^{-6}$
Fusiform gyrus L	18.0	$2 x10^{-6}$	0.000014	bvFTD < CBS P= $3 x10^{-6}$, bvFTD < PSP P=$5 x10^{-6}$
Subgenual frontal cortex R	17.7	$2 x10^{-6}$	0.000015	bvFTD < CBS P= $3 x10^{-6}$ bvFTD < PSP P= $6 x10^{-6}$
Cingulate gyrus anterior part R	17.3	$2 x10^{-6}$	0.000017	bvFTD < CBS P= $5 x10^{-6}$, bvFTD < PSP P= $6 x10^{-6}$
Anterior temporal lobe medial part R	17.1	$3 x10^{-6}$	0.000018	bvFTD < CBS P= $2 x10^{-6}$, bvFTD < PSP P= $3 x10^{-5}$
Inferior frontal gyrus R	15.9	$5 x10^{-6}$	0.000032	bvFTD < CBS P= $4 x10^{-6}$, bvFTD < PSP P= $4 x10^{-5}$
Presubgenual frontal cortex L	15.8	$6 x10^{-6}$	0.000032	bvFTD < CBS P= $9 x10^{-6}$, bvFTD < PSP P= $2 x10^{-5}$
Anterior temporal lobe medial part L	15.4	$7 x10^{-6}$	0.000039	bvFTD < CBS P=$1 x10^{-5}$, bvFTD < PSP P= $3 x10^{-5}$
Middle and inferior temporal gyrus L	15.1	$9 x10^{-6}$	0.000042	bvFTD < CBS P= $1 x10^{-5}$, bvFTD < PSP P= $3 x10^{-5}$
Medial orbital gyrus L	14.9	$1 x10^{-5}$	0.000044	bvFTD < CBS P= $9x10^{6}$, bvFTD < PSP P= $5 x10^{-5}$
Cingulate gyrus posterior part L	14.5	0.000013	0.000055	bvFTD < PSP P= $1 x10^{-5}$, bvFTD < CBS P= $6 x10^{-5}$
Insula L	14.3	0.000014	0.000058	bvFTD < CBS P= $8 x10^{-6}$, bvFTD < PSP P= 0.0003
Fusiform gyrus R	14.0	0.000017	0.000067	bvFTD < CBS P= $3 x10^{-5}$, bvFTD < PSP P= $5 x10^{-5}$
Superior frontal gryus L	13.9	0.000018	0.000067	bvFTD < CBS P= $4 x10^{-5}$, bvFTD < PSP P= $3 x10^{-5}$
Superior temporal gyrus anterior part L	13.7	0.00002	0.000072	bvFTD < CBS P= $3 x10^{-5}$, bvFTD < PSP P= $6 x10^{-5}$
Middle frontal gyrus R	13.7	0.000021	0.000072	bvFTD < CBS P= $3 x10^{-5}$, bvFTD < PSP P= $6 x10^{-5}$

Superior temporal gyrus central part R	13.6	0.000022	0.000072	bvFTD < CBS P= 1 x10^{-5},<br>bvFTD < PSP P= 0.0003
Superior temporal gyrus anterior part R	13.4	0.000025	0.00008	bvFTD < CBS P= 2 x10^{-5},<br>bvFTD < PSP P= 0.0002
Hippocampus R	13.2	0.000029	0.000087	bvFTD < CBS P= x10^{-5},<br>bvFTD < PSP P= 6 x10^{-5}
Posterior orbital gyrus R	12.9	0.000035	0.0001	bvFTD < CBS P= 4 x10^{-5},<br>bvFTD < PSP P= 0.0001
Superior frontal gyrus R	12.8	0.000038	0.00011	bvFTD < CBS P= 6 x10^{-5},<br>bvFTD < PSP P= 7.x10^{-5}
Parahippocampal and ambient gyri R	12.5	0.000047	0.00013	bvFTD < CBS P= 2 x10^{-5},<br>bvFTD < PSP P= 0.0009
Lateral orbital gyrus R	12.3	0.00005	0.00013	bvFTD < CBS P= 5 x10^{-5},<br>bvFTD < PSP P= 0.0002
Cingulate gyrus posterior part R	12.3	0.000051	0.00013	bvFTD < PSP P= 5 x10^{-5},<br>bvFTD < CBS P= 0.0002
Inferior frontal gyrus L	11.6	0.000079	0.0002	bvFTD < CBS P= 7x10^{-5},<br>bvFTD < PSP P= 0.0003
Posterior orbital gyrus L	11.5	0.000088	0.00021	bvFTD < CBS P= 8x10^{-5},<br>bvFTD < PSP P= 0.0003
Lingual gyrus L	11.4	0.000093	0.00022	bvFTD < CBS P= 7x10^{-5},<br>bvFTD < PSP P= 0.0005
Posterior temporal lobe L	11.3	0.000098	0.00022	bvFTD < CBS P= 0.0001,<br>bvFTD < PSP P= 0.0003
Hippocampus L	10.4	0.00018	0.00039	bvFTD < CBS P= 0.0003,<br>bvFTD < PSP P= 0.0003
Middle frontal gyrus L	9.4	0.00037	0.00079	bvFTD < CBS P= 0.0006,<br>bvFTD < PSP P= 0.0005
Anterior orbital gyrus L	9.1	0.00045	0.00091	bvFTD < CBS P= 0.0005,<br>bvFTD < PSP P= 0.0011
Thalamus R	9.1	0.00045	0.00091	bvFTD < CBS P= 0.0004,<br>bvFTD < PSP P= 0.048,<br>CBS > PSP P= 0.043
Anterior orbital gyrus R	8.7	0.00062	0.0012	bvFTD < CBS P= 0.0005,<br>bvFTD < PSP P= 0.003
Anterior temporal lobe lateral part L	8.4	0.00074	0.0014	bvFTD < CBS P= 0.0008,<br>bvFTD < PSP P= 0.002
Superior parietal gyrus L	8.2	0.00087	0.0016	bvFTD < PSP P= 0.0005,<br>bvFTD < CBS P= 0.011
Lateral orbital gyrus L	8.1	0.00093	0.0017	bvFTD < CBS P= 0.001,<br>bvFTD < PSP P= 0.002
Superior temporal gyrus central part L	7.9	0.0011	0.0019	bvFTD < CBS P= 0.001,<br>bvFTD < PSP P= 0.003
Pons	7.8	0.0012	0.0021	bvFTD < CBS P= 0.0009,<br>bvFTD < PSP P= 0.005
Pallidum L	7.7	0.0013	0.0022	bvFTD < CBS P= 0.002,<br>CBS > PSP P= 0.017
Midbrain	7.1	0.002	0.0033	bvFTD < CBS P= 0.002,<br>CBS > PSP P= 0.042
Postcentral gyrus L	6.5	0.0032	0.0052	bvFTD < PSP P= 0.002,<br>bvFTD < CBS P= 0.019

Posterior temporal lobe R	6.3	0.0039	0.0063	bvFTD < CBS P= 0.0045, bvFTD < PSP P= 0.007
Inferiolateral remainder of parietal lobe R	6.2	0.0042	0.0066	bvFTD < PSP P= 0.004, bvFTD < CBS P= 0.012
Nucleus Accumbens R	6.0	0.0048	0.0075	bvFTD < CBS P= 0.005, bvFTD < PSP P= 0.009
Thalamus L	5.9	0.0051	0.0077	bvFTD < CBS P= 0.003, bvFTD > PSP P= 0.043
Amygdala R	5.8	0.0055	0.0082	bvFTD < CBS P= 0.007, bvFTD < PSP P= 0.008
Pallidum R	5.4	0.0075	0.011	bvFTD < CBS P= 0.023, CBS > PSP P= 0.021
Subcallosal area R	5.4	0.0076	0.011	bvFTD < CBS P= 0.006, bvFTD < PSP P= 0.022
Postcentral gyrus R	5.3	0.0085	0.012	bvFTD < CBS P= 0.011, bvFTD < PSP P= 0.011
Precentral gyrus L	5.1	0.0096	0.013	bvFTD < PSP P= 0.007
Putamen R	4.9	0.012	0.016	bvFTD < CBS P= 0.008
Lateral remainder of occipital lobe L	4.9	0.012	0.016	bvFTD < PSP P= 0.0134, bvFTD < CBS P= 0.018
Caudate Nucleus L	4.8	0.013	0.017	bvFTD < CBS P= 0.01
Inferiolateral remainder of parietal lobe L	4.7	0.014	0.018	bvFTD < PSP P= 0.013 ,bvFTD < CBS P= 0.028
Superior parietal gyrus R	4.7	0.014	0.018	bvFTD < PSP P= 0.01
Precentral gyrus R	4.6	0.014	0.018	bvFTD < PSP P= 0.012, bvFTD < CBS P= 0.038
Cerebellum gm L	4.6	0.015	0.019	bvFTD < PSP P= 0.016, bvFTD < CBS P= 0.024
Caudate Nucleus R	4.5	0.016	0.019	bvFTD < CBS P= 0.012
Nucleus Accumbens L	4.5	0.017	0.02	bvFTD < PSP P= 0.015, bvFTD < CBS P= 0.034
Putamen L	4.5	0.017	0.02	bvFTD < CBS P= 0.013
Cuneus R	4.5	0.017	0.02	bvFTD < CBS P= 0.022, bvFTD < PSP P= 0.021
Cuneus L	4.3	0.019	0.021	bvFTD < CBS P= 0.015
Substantia nigra L	4.3	0.019	0.021	CBS > PSP P= 0.018
Subcallosal area L	4.3	0.019	0.021	bvFTD < CBS P= 0.017 bvFTD < PSP P= 0.040
Amygdala L	3.5	0.04	0.043	bvFTD < CBS P= 0.050, bvFTD < PSP P= 0.045
Lingual gyrus R	3.4	0.043	0.047	bvFTD < CBS P= 0.046
Lateral remainder of occipital lobe R	3.1	0.057	0.061	
Substantia nigra R	2.4	0.1	0.11	
Cerebellum dentate L	1.9	0.17	0.17	
Cerebellum gm R	1.7	0.2	0.21	
Cerebellum dentate R	0.8	0.47	0.48	
Medulla	0.6	0.55	0.55	

Post-hoc tests only performed where there is an uncorrected group difference in an ANCOVA with age and sex as covariates of no interest. Only significant results after Tukey adjustment of p-values with two-sided testing are shown.

Supplementary Table 5. Regional differences in grey matter volumes between patient groups

Region	F value	P	FDR P	*Post-hoc* tests
Nucleus Accumbens R	13.5	0.000022	0.0016	bvFTD < CBS P= $1x10^{-5}$<br>bvFTD < PSP P= 0.0008
Amygdala R	12.5	0.00004	0.0016	bvFTD < CBS P= 0.0004<br>bvFTD < PSP P= $3x10^{-5}$
Medial orbital gyrus R	10.9	0.00012	0.0024	bvFTD < CBS P= 0.0002<br>bvFTD < PSP P= 0.0003
Caudate Nucleus R	10.6	0.00015	0.0024	bvFTD < CBS P= 0.0004<br>bvFTD < PSP P= 0.0002
Medial orbital gyrus L	10.5	0.00015	0.0024	bvFTD < CBS P= 0.0007<br>bvFTD < PSP P= 0.0002
Anterior orbital gyrus R	10.0	0.00023	0.0028	bvFTD < CBS P= 0.001<br>bvFTD < PSP P= 0.0002
Subcallosal area R	9.9	0.00025	0.0028	bvFTD < CBS P= 0.0006<br>bvFTD < PSP P= 0.0004
Parahippocampal and ambient gyri R	9.6	0.0003	0.003	bvFTD < CBS P= 0.013<br>bvFTD < PSP P= 0.0002
Superior frontal gryus L	9.3	0.00038	0.0031	bvFTD < CBS P= 0.019<br>bvFTD < PSP P= 0.0002
Posterior orbital gyrus L	9.3	0.00039	0.0031	bvFTD < CBS P= 0.009<br>bvFTD < PSP P= 0.0002
Inferior frontal gyrus L	8.6	0.00063	0.0045	bvFTD < CBS P= 0.0009<br>bvFTD < PSP P= 0.001
Insula L	8.2	0.00086	0.0049	bvFTD < PSP P= 0.0007
Superior parietal gyrus L	8.2	0.00086	0.0049	bvFTD < PSP P= 0.0008
Nucleus Accumbens L	8.2	0.00088	0.0049	bvFTD < CBS P= 0.001<br>bvFTD < PSP P= 0.002
Cingulate gyrus posterior part R	7.9	0.0011	0.005	bvFTD < PSP P= 0.001
Middle and inferior temporal gyrus R	7.9	0.0011	0.005	bvFTD < CBS P= 0.002<br>bvFTD < PSP P= 0.002
Anterior temporal lobe lateral part R	7.8	0.0011	0.005	bvFTD < CBS P= 0.0008<br>bvFTD < PSP P= 0.009
Middle frontal gyrus L	7.7	0.0012	0.005	bvFTD < CBS P= 0.006<br>bvFTD < PSP P= 0.001
Posterior orbital gyrus R	7.7	0.0012	0.005	bvFTD < CBS P= 0.006<br>bvFTD < PSP P= 0.001
Caudate Nucleus L	7.2	0.0018	0.0071	bvFTD < CBS P= 0.004<br>bvFTD < PSP P= 0.002
Anterior temporal lobe medial part R	7.0	0.0021	0.008	bvFTD < CBS P= 0.010<br>bvFTD < PSP P= 0.002
Hippocampus R	6.9	0.0024	0.0085	bvFTD < CBS P= 0.004<br>bvFTD < PSP P= 0.003
Superior temporal gyrus anterior part L	6.7	0.0027	0.0092	bvFTD < CBS P= 0.020<br>bvFTD < PSP P= 0.002

Cingulate gyrus anterior part R	6.6	0.0029	0.0092	bvFTD < CBS P= 0.002<br>bvFTD < PSP P= 0.025
Anterior orbital gyrus L	6.6	0.0029	0.0092	bvFTD < CBS P= 0.009<br>bvFTD < PSP P= 0.003
Insula R	6.4	0.0035	0.011	bvFTD < CBS P= 0.017<br>bvFTD < PSP P= 0.0026
Superior frontal gyrus R	6.2	0.0039	0.011	bvFTD < CBS P= 0.043<br>bvFTD < PSP P= 0.003
Lateral orbital gyrus L	6.1	0.0042	0.012	bvFTD < CBS P= 0.021<br>bvFTD < PSP P= 0.003
Parahippocampal and ambient gyri L	6.1	0.0044	0.012	bvFTD < PSP P= 0.003
Subcallosal area L	6.0	0.0045	0.012	bvFTD < CBS P= 0.007<br>bvFTD < PSP P= 0.006
Midbrain	5.6	0.0063	0.016	bvFTD < PSP P= 0.12<br>CBS > PSP P= 0.007
Anterior temporal lobe lateral part L	5.5	0.0069	0.017	bvFTD - CBS P= 0.008<br>bvFTD - PSP P= 0.012
Inferior frontal gyrus R	5.4	0.0077	0.018	bvFTD < CBS P= 0.041<br>bvFTD < PSP P= 0.006
Middle frontal gyrus R	5.0	0.01	0.024	bvFTD < PSP P= 0.0073
Middle and inferior temporal gyrus L	5.0	0.011	0.024	bvFTD < CBS P= 0.012<br>bvFTD < PSP P= 0.018
Precentral gyrus R	4.9	0.012	0.026	CBS < PSP P= 0.019
Putamen L	4.7	0.014	0.029	bvFTD < PSP P= 0.014
Posterior temporal lobe L	4.5	0.016	0.033	bvFTD < PSP P= 0.012
Precentral gyrus L	4.3	0.019	0.038	CBS < PSP P= 0.022
Amygdala L	4.3	0.019	0.038	bvFTD < PSP P= 0.015
Cerebellum dentate L	4.2	0.021	0.041	CBS > PSP P= 0.047
Anterior temporal lobe medial part L	4.1	0.023	0.044	bvFTD < PSP P= 0.019
Superior temporal gyrus anterior part R	4.0	0.024	0.045	bvFTD < PSP P= 0.020
Posterior temporal lobe R	4.0	0.025	0.045	bvFTD <CBS P= 0.29<br>bvFTD < PSP P= 0.022
Cingulate gyrus anterior part L	3.9	0.028	0.049	bvFTD < CBS P= 0.039<br>bvFTD < PSP P= 0.033
Hippocampus L	3.6	0.034	0.057	bvFTD < PSP P= 0.034
Postcentral gyrus L	3.6	0.034	0.057	
Postcentral gyrus R	3.6	0.036	0.059	
Lateral orbital gyrus R	3.2	0.048	0.078	bvFTD < PSP P= 0.038
Cingulate gyrus posterior part L	2.6	0.082	0.13	
Superior parietal gyrus R	2.6	0.083	0.13	
Inferiolateral remainder of parietal lobe L	2.5	0.089	0.13	
Cerebellum dentate R	2.4	0.097	0.15	
Pons	2.3	0.11	0.16	

Thalamus R	2.1	0.14	0.19	
Fusiform gyrus L	1.9	0.16	0.22	
Fusiform gyrus R	1.9	0.16	0.22	
Subgenual frontal cortex R	1.9	0.17	0.22	
Inferiolateral remainder of parietal lobe R	1.9	0.17	0.22	
Presubgenual frontal cortex L	1.8	0.18	0.24	
Pallidum R	1.7	0.19	0.24	
Subgenual frontal cortex L	1.7	0.19	0.24	
Substantia nigra L	1.7	0.19	0.24	
Lateral remainder of occipital lobe L	1.6	0.21	0.25	
Superior temporal gyrus central part R	1.5	0.23	0.28	
Lingual gyrus L	1.3	0.28	0.33	
Pallidum L	1.3	0.29	0.35	
Putamen R	1.2	0.31	0.36	
Lateral remainder of occipital lobe R	1.2	0.32	0.36	
Medulla	1.1	0.33	0.37	
Substantia nigra R	1.1	0.35	0.39	
Superior temporal gyrus central part L	0.6	0.57	0.63	
Presubgenual frontal cortex L	0.5	0.6	0.65	
Thalamus L	0.4	0.67	0.72	
Cuneus L	0.3	0.74	0.78	
Cerebellum gm R	0.3	0.78	0.81	
Lingual gyrus R	0.2	0.8	0.82	
Cuneus R	0.2	0.82	0.83	
Cerebellum gm L	0.0	0.99	0.99	

Post-hoc tests only performed where there is an uncorrected group difference in an ANCOVA with age and sex as covariates of no interest Only significant results after Tukey adjustment of p-values with two-sided testing are shown.

Supplementary Table 6. Fixed effects for relationship between synaptic density and weighted degree including grey matter volume in patients

	Std Beta	SE	T value	P
**Intercept**	0	0.10	-0.02	0.98
**UCB-J**	0.18	0.03	6.2	$1x10^{-9}$
**Grey matter vol**	0.12	0.03	3.9	0.0001
**Age**	-0.15	0.09	-1.6	0.12
**Mean DVARS**	0.16	0.09	1.7	0.10
**Sex**	0.04	0.09	0.37	0.71
**Cortical/subcortical**	0.01	0.04	0.16	0.87

P values of the fixed effects were calculated with two-sided testing using Satterthwaite's method.

DVARS: the spatial standard deviation of successive difference images

Supplementary Table 7. Final model for stepwise regression for ACE-R total

	Std Beta	Std Error	T value	P
**Intercept**	-0.04	0.11	-0.3	0.74
**fMRI 4**	0.06	0.12	0.49	0.63
**fMRI 5**	0.07	0.13	0.54	0.59
**UCB-J 2**	0.40	0.11	3.7	0.0007
**UCB-J 4**	0.55	0.13	4.2	0.0001
**UCB-J 5**	0.14	0.16	0.91	0.37
**Mean DVARS**	0.09	0.11	0.82	0.42
**Age**	0.13	0.13	1.0	0.30
**Sex**	0.13	0.11	1.3	0.22
**fMRI 4 * UCB-J 4**	0.28	0.10	2.9	0.005
**fMRI 5 * UCB-J 5**	0.18	0.09	2.0	0.053

P values for the final model were calculated in a linear model with two-sided testing. ACE-R: Addenbrooke's Cognitive Examination-Revised, DVARS the spatial standard deviation of successive difference images.

Supplementary Table 8. Final model for stepwise regression for PSPRS total

	Std Beta	Std Error	T value	P
**Intercept**	0.18	0.13	0.7	0.51
**UCB-J 1**	-0.46	0.15	-3.1	0.003
**UCB-J 5**	-0.13	0.19	-0.7	0.50
**fMRI 5**	-0.25	0.15	-1.7	0.09
**Mean DVARS**	-0.06	0.12	-0.4	0.68
**Age**	-0.15	0.14	-1.0	0.31
**Sex**	-0.08	0.14	-0.6	0.56
**fMRI 5 * UCB-J 5**	-0.22	0.1	-2.0	0.048

P values for the final model were calculated in a linear model with two-sided testing. PSPRS - Progressive supranuclear palsy rating scale, DVARS the spatial standard deviation of successive difference images

Supplementary Table 9. Final model for stepwise regression for ACE-R total using connectivity scores outside UCB-J BP_{ND} component

	Std Beta	Std Error	T value	P
**Intercept**	-0.02	0.11	-0.23	0.81
**fMRI 4**	0.05	0.12	0.41	0.68
**fMRI 5**	0.03	0.13	0.22	0.83
**UCB-J 2**	0.39	0.11	3.6	0.0009
**UCB-J 4**	0.53	0.13	4.1	0.0002
**UCB-J 5**	0.15	0.16	0.95	0.35
**Mean DVARS**	0.11	0.11	0.92	0.36
**Age**	0.13	0.13	1.0	0.30
**Sex**	0.13	0.11	1.2	0.23
**fMRI 4 * UCB-J 4**	0.28	0.09	3.0	0.005
**fMRI 5 * UCB-J 5**	0.16	0.09	1.9	0.067

P values for the final model were calculated in a linear model with two-sided testing. ACE-R: Addenbrooke's Cognitive Examination-Revised, DVARS the spatial standard deviation of successive difference images.

Supplementary Table 10. Final model for stepwise regression for PSPRS total using connectivity scores outside UCB-J BP_{ND} component

	Std Beta	Std Error	T value	P
**Intercept**	0	0.12	0	1
**UCB-J 1**	-0.51	0.13	-4.0	0.0002
**Mean DVARS**	-0.07	0.12	-0.6	0.54
**Age**	-0.02	0.13	-0.18	0.86
**Sex**	-0.07	0.13	-0.6	0.57

P values for the final model were calculated in a linear model with two-sided testing. PSPRS - Progressive supranuclear palsy rating scale, DVARS the spatial standard deviation of successive difference images

Supplementary references

1. Di, X. & Biswal, And Alzheimer's Disease Neu, B. B. Metabolic Brain Covariant Networks as Revealed by FDG-PET with Reference to Resting-State fMRI Networks. *Brain Connectivity* **2**, 275–283 (2012).
