# Supplementary material for: Synaptic density affects clinical severity via network dysfunction in syndromes associated with frontotemporal lobar degeneration
Source: Nat Commun. 2023 Dec 20;14:8458. doi: 10.1038/s41467-023-44307-7 (PMC10730886; doi:10.1038/s41467-023-44307-7)
Supplement: Supplementary file 3 — Reporting Summary [file 41467_2023_44307_MOESM3_ESM.pdf]

nature portfolio

Corresponding author(s): David Whiteside
Last updated by author(s): Nov 25, 2023

Reporting Summary

Nature Portfolio wishes to improve the reproducibility of the work that we publish. This form provides structure for consistency and transparency in reporting. For further information on Nature Portfolio policies, see our Editorial Policies and the Editorial Policy Checklist.

Statistics

For all statistical analyses, confirm that the following items are present in the figure legend, table legend, main text, or Methods section.

n/a	Confirmed	
☐	☒	The exact sample size (*n*) for each experimental group/condition, given as a discrete number and unit of measurement
☐	☒	A statement on whether measurements were taken from distinct samples or whether the same sample was measured repeatedly
☐	☒	The statistical test(s) used AND whether they are one- or two-sided<br>*Only common tests should be described solely by name; describe more complex techniques in the Methods section.*
☐	☒	A description of all covariates tested
☐	☒	A description of any assumptions or corrections, such as tests of normality and adjustment for multiple comparisons
☐	☒	A full description of the statistical parameters including central tendency (e.g. means) or other basic estimates (e.g. regression coefficient) AND variation (e.g. standard deviation) or associated estimates of uncertainty (e.g. confidence intervals)
☐	☒	For null hypothesis testing, the test statistic (e.g. *F*, *t*, *r*) with confidence intervals, effect sizes, degrees of freedom and *P* value noted<br>*Give P values as exact values whenever suitable.*
☒	☐	For Bayesian analysis, information on the choice of priors and Markov chain Monte Carlo settings
☒	☐	For hierarchical and complex designs, identification of the appropriate level for tests and full reporting of outcomes
☐	☒	Estimates of effect sizes (e.g. Cohen's *d*, Pearson's *r*), indicating how they were calculated

Our web collection on statistics for biologists contains articles on many of the points above.

Software and code

Policy information about availability of computer code

Data collection	No custom software was used for data collection
Data analysis	All software used is publically available<br>PET image preprocessing and analysis:<br>Emission image series were aligned using SPM12 (www.fil.ion.ucl.ac.uk/spm/software/spm12/)<br>Image normalization for voxelwise analysis was performed using Advanced Normalization Tools (ANTS) version 2.1.0<br>Source based synaptometry used the GIFT toolbox version 4.0c (https://trendscenter.org/software/gift/)<br>Structural MRI image analysis:<br>Volumetric T1-weighted MRI images grey matter volumes were extracted for regions of the Hammersmith Atlas using SPM12 (SPM12 v7771)<br>Total intracranial volume was calculated via direct segmentation using Sequence Adaptive Multimodal SEGmentation<br>NODDI imaging preprocessing and analysis:<br>Preprocessing was performed using tools from FSL.<br>Orientation Dispersion Index values were derived using the Microstructural Diffusion Toolbox<br>Image normalization for voxelwise analysis was performed using Advanced Normalization Tools (ANTS) version 2.1.0<br>Functional MRI analysis:<br>Preprocessing used tools available from FSL version 6.0.4 as described below<br>Wavelet despiking was performed using the Brain Wavelet toolbox v2.0<br>Weighted degree was derived using Maybrain https://github.com/RittmanResearch/maybrain and NetworkX (https://networkx.org/)<br>Connectivity of UCB-J components was calculated using FSL's dual regression function

Statistical significance for voxelwise comparisons used FSL's randomise tool and FSL's Permutation Analysis of Linear Models version alpha-119
Statistical analysis and visualisation was carried out in R Version 4.1.2
Spatial autocorrelation preserving null models and related statistical testing were derived in the neuromaps toolbox (https://github.com/netneurolab/neuromaps)
Code for preprocessing and analysis is available at https://github.com/djw216/synaptic-pet-fmri

For manuscripts utilizing custom algorithms or software that are central to the research but not yet described in published literature, software must be made available to editors and reviewers. We strongly encourage code deposition in a community repository (e.g. GitHub). See the Nature Portfolio guidelines for submitting code & software for further information.

Data

Policy information about availability of data

All manuscripts must include a data availability statement. This statement should provide the following information, where applicable:

- Accession codes, unique identifiers, or web links for publicly available datasets
- A description of any restrictions on data availability
- For clinical datasets or third party data, please ensure that the statement adheres to our policy

Derived imaging data, including those required to generate Figures 1-3 and supplementary figures, are provided in the Source Data file. These data have also been deposited in a Figshare repository (https://doi.org/10.6084/m9.figshare.24188580).91 Access to raw imaging data and linked clinical data may be available via an agreed third party Secure Research Environment or by transfer under a material/data transfer agreement, subject to conditions required to comply with participant consent and data protection regulations. Requests should be addressed to the senior author in the first instance. Initial responses to queries or requests for data, determining the need for any material/data transfer agreement, will be provided within a month of a request being made. The n30r83 Hammersmith atlas was modified for regional analysis and is publicly available at http://brain-development.org. The Brainnetome Atlas (https://atlas.brainnetome.org/) used for ensuring results are independent of parcellation in this study is publicly available.

Research involving human participants, their data, or biological material

Policy information about studies with human participants or human data. See also policy information about sex, gender (identity/presentation), and sexual orientation and race, ethnicity and racism.

Reporting on sex and gender	Self-reported sex for each group is shown in Table 1. Sex was included as a covariate of no interest in all between-subject analyses
Reporting on race, ethnicity, or other socially relevant groupings	We did not perform analyses by race/ethnicity or other social grouping.
Population characteristics	As described in table 1, this study consisted of 24 controls, 29 participants with progressive supranuclear palsy, 16 participants with amyloid negative corticobasal syndrome, and 10 participants with behavioural variant FTD
Recruitment	Participants with neurodegenerative diseases were recruited from tertiary clinics at the Cambridge Centre for Parkinson-plus, the Cambridge Centre for Frontotemporal Dementia, and National Hospital for Neurology and Neurosurgery at Queen Square, London. 24 similarly-aged healthy volunteers were recruited from the UK National Institute for Health Research Join Dementia Research (JDR) register. Potential participants with corticobasal syndrome were excluded if they had a positive amyloid PET scan (as characterized by a cortical [11C]PiB SUVR <1.21, obtained by converting the Centiloid cut-off of 19 to SUVR using the Centiloid-to-SUVR transformation)). Participants were initially screened via telephone, using the following exclusion criteria: a current or recent history of cancer within the last 5 years, concurrent use of the medication levetiracetam, any contraindications to undergoing an MRI, a history of ischaemic or haemorrhagic stroke evident on the MRI from the clinic, and any severe physical illness or co-morbidity that could limit their ability to fully participate in the study. Twenty-three participants who passed initial screening were not included in this study due to positive Alzheimer's biomarkers (all CBS, n=9) or due to failure to complete all scanning sessions (n=14: Control n=8, bvFTD n=1, CBS n=1, PSP n=4).<br>We recognise that recruitment to observational and biomarker studies can be prone to self-selection biases, and so the study participants may not reflect all demographic characteristics of the wider population. This may be exacerbated by the challenge of multi-modal imaging studies with long acquisition times. To limit the impact of these biases we utilised the syndromic variability in frontotemporal lober degeneration syndromes to capture differences in synaptic density. Further replication work is needed to test whether our findings are observed in different populations.
Ethics oversight	The research protocol (18/EE/0059) was approved by the Cambridge Research Ethics Committee and the Administration of Radioactive Substances Advisory Committee.

Note that full information on the approval of the study protocol must also be provided in the manuscript.

Field-specific reporting

Please select the one below that is the best fit for your research. If you are not sure, read the appropriate sections before making your selection.

☒ Life sciences ☐ Behavioural & social sciences ☐ Ecological, evolutionary & environmental sciences

For a reference copy of the document with all sections, see nature.com/documents/nr-reporting-summary-flat.pdf

Life sciences study design

All studies must disclose on these points even when the disclosure is negative.

Sample size	Data was collected as part of studies on synaptic density in syndromes associated with frontotemporal lobar degeneration (DOI:10.1002/mds.28188 and DOI:10.1002/ana.26543). These studies have shown large effect sizes in differentiating people with neurodegenerative diseases from healthy controls and in relation to clinical-rated and carer-based markers of severity (Pearson's R 0.5-0.75), suggesting our sample size was suitable to detect relationships of interest. Although no sample size calculation was performed specifically for this study we were powered to detect a correlation between connectivity and synaptic density of approximately r=0.4 using averaged values across participants.
Data exclusions	Potential participants with corticobasal syndrome were excluded if they had a positive amyloid PET scan (as characterized by a cortical [11C] PiB SUVR <1.21, obtained by converting the Centiloid cut-off of 19 to SUVR using the Centiloid-to-SUVR transformation)), in order to decrease pathological heterogeneity. Participants needed to complete both the PET and fMRI scanning session to be included.
Replication	This study was conducted in a single cohort, with further studies needed to demonstrate replication of findings
Randomization	Age. sex and in-scanner motion were included as covariates of no interest in between-group analyses as covariates of no interest
Blinding	Blinding was not relevant to this observational study

Reporting for specific materials, systems and methods

We require information from authors about some types of materials, experimental systems and methods used in many studies. Here, indicate whether each material, system or method listed is relevant to your study. If you are not sure if a list item applies to your research, read the appropriate section before selecting a response.

Materials & experimental systems

n/a	Involved in the study	
☒	☐	Antibodies
☒	☐	Eukaryotic cell lines
☒	☐	Palaeontology and archaeology
☒	☐	Animals and other organisms
☐	☒	Clinical data
☒	☐	Dual use research of concern
☒	☐	Plants

Methods

n/a	Involved in the study	
☒	☐	ChIP-seq
☒	☐	Flow cytometry
☐	☒	MRI-based neuroimaging

Clinical data

Policy information about clinical studies

All manuscripts should comply with the ICMJEguidelines for publication of clinical research and a completedCONSORT checklist must be included with all submissions.

Clinical trial registration	This study did not require clinical trial registration - Research protocol approved by Cambridge Research Ethics Committee (SENDER study - 18/EE/0059)
Study protocol	The study protocol for SENDER is available from the senior authors
Data collection	Participants with neurodegenerative diseases were recruited from tertiary clinics at the Cambridge Centre for Parkinson-plus, the Cambridge Centre for Frontotemporal Dementia, and National Hospital for Neurology and Neurosurgery at Queen Square, London. All imaging was performed at the Wolfson Brain Imaging Centre, University of Cambridge. Data collection took place between June 2019 and May 2022
Outcomes	The key hypothesis of our study was that functional connectivity would relate to synaptic density in neurodegenerative diseases. We tested this using regional and voxelwise methods with graph metric analysis and seed-based dual regression respectively.

Magnetic resonance imaging

Experimental design

Design type	Resting state
Design specifications	N/A
Behavioral performance measures	N/A

Acquisition

Imaging type(s)	Functional and structural imaging
Field strength	3
Sequence & imaging parameters	Functional MRI was performed with 3-Tesla Siemens Prisma (Siemens Healthcare) using echo-planar imaging sensitive to the blood-oxygen-level-dependent signal (TR 2.5 secs, TE 30ms, whole brain acquisition, 3x3x3.5mm voxels, 200 volumes). High resolution T1-weighted Magnetization Prepared Rapid Gradient Echo (MPRAGE) structural images (TR 2, TE 2.93ms, voxel size 1.1mm isotropic) were acquired during the same session.
Area of acquisition	Whole brain scanning

Diffusion MRI ☒ Used ☐ Not used

Parameters: Diffusion scans were acquired on Siemens Magnetom Prisma scanner (TE = 75.6 ms, TR = 2.4s , slice thickness = 1.75 mm, 98 directions, 104 slices, bvals = 300, 1000, 2000).

Preprocessing

Preprocessing software	fMRI preprocessing followed the FSL pipeline (version 6.0.4) with the addition of wavelet despiking.. T1 structural images were cropped to remove non-brain tissue followed by brain extraction using FSL's Brain Extraction Tool. We then used FSL's FEAT with the following steps: motion correction using MCFLIRT; spatial smoothing using a Gaussian kernel of 5mm full-width at half maximum; grand-mean intensity normalisation of the 4D dataset by a single multiplicative factor; and 100Hz high-pass temporal filtering. Structured artefacts were removed using independent component analysis denoising using FSL's MELODIC together with FIX. FIX was hand-trained using a set of 20 subjects. For dual regression analysis normalized data was further smoothed with a 6mm FWHM Gaussian kernel.
Normalization	Registration to high resolution structural images and from structural images to MNI space was carried out using FSL's FLIRT. Registration from high resolution structural to MNI space was then further refined using FNIRT nonlinear registration.
Normalization template	FSL's MNI 152 2mm isotropic template
Noise and artifact removal	FIX-ICA based denoising followed by wavelet despiking
Volume censoring	No volume censoring

Statistical modeling & inference

Model type and settings	N/A
Effect(s) tested	N/A

Specify type of analysis: ☒ Whole brain ☐ ROI-based ☐ Both

Statistic type for inference (See Eklund et al. 2016)	The association between [11C]UCB-J BP loadings values and functional covariance per component was assessed in a general linear model with covariatesusing threshold free cluster enhancement with 5000 permutations using FSL's randomise tool.
Correction	For voxel-wise analysis family-wise error correction was performed. For derived scores (i.e. connectivity scores versus [11C] UCB-J BP loadings) false discovery rate correction was performed.

Models & analysis

n/a	Involved in the study
☐	☒ Functional and/or effective connectivity
☐	☒ Graph analysis
☒	☐ Multivariate modeling or predictive analysis

Functional and/or effective connectivity	Spatial covariance with [11C]UCB-J BP was derived using the dual regression approach.
Graph analysis	Pearson correlations were calculated between nodes for a sub-parcellation of the Hammersmith Atlas, followed by Fisher's r-to-Z transformation. We then calculated mean weighted degree across the sub-parcellations for each Hammersmith atlas region to compare with regional [11C]UCB-J BPND
